# Yes, there is a medial nucleus of the trapezoid body in humans

**DOI:** 10.3389/fnana.2015.00035

**Published:** 2015-03-31

**Authors:** Randy J. Kulesza, Benedikt Grothe

**Affiliations:** ^1^Department of Anatomy, Auditory Research Center, Lake Erie College of Osteopathic MedicineErie, PA, USA; ^2^Division of Neurobiology, Department Biologie II, Ludwig-Maximilians-Universität MünchenMunich, Germany

**Keywords:** auditory, brainstem, cochlear nucleus, calyx of held, superior olive

## Abstract

The medial nucleus of the trapezoid body (MNTB) is a collection of brainstem neurons that function within the ascending auditory pathway. MNTB neurons are associated with a number of anatomical and physiological specializations which make these cells especially well-equipped to provide extremely fast and precise glycinergic inhibition to its target neurons in the superior olivary complex and ventral nucleus of the lateral lemniscus. The inhibitory influence of MNTB neurons plays essentials roles in the localization of sound sources and encoding temporal features of complex sounds. The morphology, afferent and efferent connections and physiological response properties of MNTB neurons have been well-characterized in a number of laboratory rodents and some carnivores. Furthermore, the MNTB has been positively identified in all mammals examined, ranging from opossum and mice to chimpanzees. From the early 1970s through 2009, a number of studies denied the existence of the MNTB in humans and consequentially, the existence of this nucleus in the human brain has been debated for nearly 50 years. The absence of the MNTB from the human brain would negate current principles of sound localization and would require a number of novel adaptations, entirely unique to humans. However, a number of recent studies of human post-mortem tissue have provided evidence supporting the existence of the MNTB in humans. It therefore seems timely to review the structure and function of the MNTB, critically review the literature which led to the denial of the human MNTB and then review recent investigations supporting the existence of the MNTB in the human brain.

## Introduction: The MNTB Dilemma

The question whether the human, unlike other mammals, lacks a MNTB has been debated for several decades. The question is an important one because the MNTB is a major component of the mammalian SOC, a major source of glycinergic inhibition to auditory brainstem centers and MNTB neurons are associated with a number of anatomical and physiological specializations. Against profound evidence for the existence of the MNTB in humans and in the face of severe conceptual problems, many auditory scientists seem to accept its non-existence and thus do not consider its inhibitory impact on target structures, e.g., the medial and MSOs and LSOs.

**FIGURE 1:**
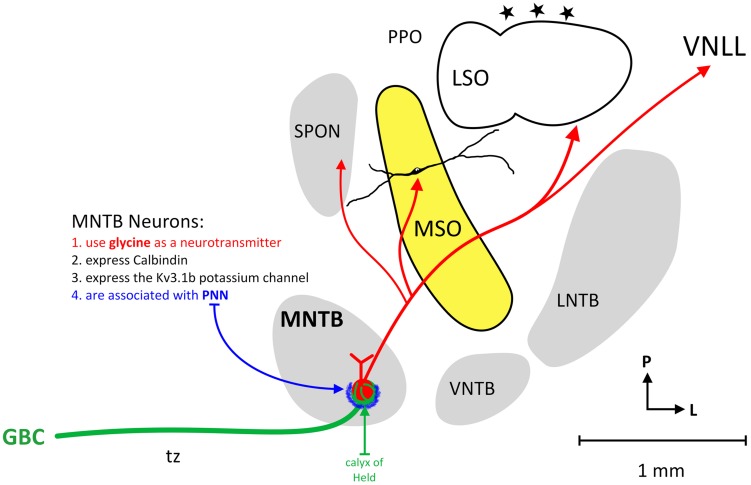
**Circuitry of the human MNTB.** The figure illustrates the nuclei that make up the human SOC and the connections of MNTB neurons. The MNTB is situated ventral (anterior) and medial to the MSO and receives its main excitatory input from the contralateral cochlear nucleus via GBCs and the calyx of Held (green line). MNTB neurons characteristically express calbindin, the Kv3.1b potassium channel and MNTB somata are associated with perineuronal nets (blue). The MNTB provides topographic, glycinergic projections to the SPON, MSO, LSO, and VNLL.

The MNTB is not the only group of neurons providing inhibition to the SOC, an important mammalian auditory brainstem center, but it is the most prominent. Principal neurons of the MNTB receive their main excitatory input from GBCs in the contralateral ventral cochlear nucleus. The GBC-MNTB pathway exhibits a number of extraordinary specialized anatomical and physiological features related to fast and high-fidelity processing of temporal information, the calyx of Held being the most prominent one. The GBCs and calyx terminals are characteristically CR (**Figure 1**; Arai et al., 1991; Resibois and Rogers, 1992; Lohmann and Friauf, 1996; Bazwinsky et al., 2005), Rab3a and VGLUT1 positive (Felmy and Schneggenburger, 2004). The principal neurons of the MNTB are distinctively CB-IR and perineuronal nets are known to ensheath the soma and primary dendrites of these neurons (**Figure 1**; Härtig et al., 2001; Hilbig et al., 2007; Schmidt et al., 2010; Blosa et al., 2013) and MNTB neurons are known to specifically express the Kv3.1b potassium channel (Wang et al., 1998).

The glutamatergic input via the famous Calyx of Held converts excitatory input, in a most precise manner, into glycinergic inhibition in its target nuclei (Moore and Caspary, 1983; Wenthold et al., 1987; von Gersdorff and Borst, 2002; **Figure 1**). Important targets of MNTB axons are neurons involved in sound localization, e.g., MSO and LSO (Zarbin et al., 1981; Moore and Caspary, 1983; Kuwabara and Zook, 1992; Grothe and Sanes, 1993; Kapfer et al., 2002). In addition, MNTB axons also target neurons that are more or less exclusively concerned with monaural temporal information, e.g., the SPN (Kuwabara et al., 1991; Sommer et al., 1993; Behrend et al., 2002; Dehmel et al., 2002; Kulesza et al., 2007), what appear to be MSO neurons in some highly specialized bats (Grothe, 2000), and the monaural nuclei of the LL (Spangler et al., 1985; Sommer et al., 1993; Smith et al., 1998). These monaural nuclei appear to be important for processing temporal features of sounds such as amplitude modulations, sound onset and/or offset or gaps in or between sounds (Grothe, 1994; Behrend et al., 2002; Kadner and Berrebi, 2008; Kopp-Scheinpflug et al., 2011; Felix et al., 2014). In some auditory brainstem neurons, the pharmacological blockade of MNTB-derived inhibition significantly alters timing of action potentials and basic response properties. For instance, the LSO (Moore and Caspary, 1983) but also many SPN (Behrend et al., 2002; Kulesza et al., 2007) and some MSO cells (again in some species of bat; Grothe, 1994) are functionally characterized by the interaction of one excitatory and one inhibitory input only.

Because of the importance of the MNTB in auditory function, particularly sound localization, it seems timely to review the history of the confusion about the human SOC and the status of the current evidence. There is a persistent belief that humans, and only humans, are missing the MNTB – although not the other major SOC components or the VNLL. An arrangement of the human SOC without an MNTB would imply that most of the processing strategies discovered in experimental mammals would require a completely different solution in the human auditory pathway. Moreover, it would be a rather unique example of a fundamental difference in the human brain compared to other terrestrial mammals ^1^. If humans were to lack an MNTB, it would generally bring into question the usefulness of animal models for the study of auditory central processing and translational approaches. Nonetheless, there is no need to postulate such phylogenetic curiosities.

## Investigations of the Human MNTB

Determining whether a cluster of neurons in the human brain is homologous with a nucleus in another species requires careful consideration of a number of objective criteria and appropriate quantitative analyses. Here, we will consider the most widely accepted identifying features of the MNTB (described above) and examine the human brainstem using five criteria:

The human MNTB should:

(1) be found in the same approximate region of the brainstem as in other species
(2) be found in the same location relative to other major nuclei and axon bundles
(3) include neurons with the same morphology as has been observed in other species
(4) have the same afferent and efferent axonal connections as has been shown in other species
(5) have the same neurochemical and functional properties as the nucleus in other species.

Determining, with certainty, that the human brainstem contains an MNTB is a challenge. First, it is impossible, using the techniques currently available, to study physiological response properties of human MNTB neurons. Second, it is nearly impossible to preserve human brainstem tissue in the same manner or quality as used for animal studies. This undoubtedly leads to deterioration of small molecules and some protein antigens. Finally, tracing the afferent and efferent connections of the human MNTB is an additional experimental challenge as such experiments can only be done in post-mortem and immersion fixed tissue.

### The Human MNTB: The Denial of its Existence and the Implications of this Claim

Many of the investigations of the human auditory brainstem left considerable doubt about the existence of the MNTB in humans, suggesting that it is absent or vestigial.

The first detailed investigation of the human SOC was published by Moore and Moore (1971), who also examined other primates. These authors utilized cresyl violet staining to investigate every seventh section (thickness = 30 μm) through a single human brainstem and found only occasional round or oval cells in the area ventral and medial to the (easily identified) MSO. They noted that these cells only occasionally formed a definable nucleus. A schematic figure of the SOC from a series of primates demonstrated the MNTB in all species examined (loris, marmoset, galago, owl monkey, macaque, and gibbon) except human. However, no images of putative MNTB neurons were shown nor was a figure of the human SOC provided. Strominger and Hurwitz (1976) examined every 10th section (thickness = 20 μm) from the brainstems of five adults, two infants and one fetus (using cresyl violet and/or luxol fast blue staining) and concluded that an “NTB” could not be defined. Notably, none of the figures in this manuscript depicted the region ventral and medial to the MSO and the reader is left only with the author’s interpretation. Strominger (1978) later went on to report some neurons “ventromedial” to the MSO which may represent the MNTB but indicated that these cells appeared cytoarchitecturally different from neurons in other species. Strominger thus concluded that the MNTB was poorly defined in human.

Moore (1987), in a further study of four human brainstems (examining every 10th or 20th tissue section [thickness = 50 μm] and using Nissl and myelin staining techniques), noted that a small number of globular neurons were found in the region where the MNTB should be located but that they did not form a nucleus. It is also stated emphatically that there was “no clearly definable nucleus of the trapezoid body” and that “no distinct cell group comparable to the trapezoid nucleus is evident in the human SOC.” The schematics of the SOC provided (Figures 8 and 9) labeled only the MSO and LSO. Again, no Nissl stained images of the SOC as a whole were shown (only Woelcke stained) and only a single high-magnification view of “periolivary” cells which were located along the rostral pole of the SOC was provided. Moore et al. (1999) later examined brainstem tissue from 12 human brains [ranging in age from 16 weeks prenatal (section thickness = 40 μm) to 17 years (section thickness = 75 μm)] with immunohistochemistry for choline acetyltransferase and calcitonin-gene-related peptide. Again, the authors stated that there was no identifiable MNTB in the human brain. Their schematics of the SOC include the MSO, LSO and periolivary regions. Again, no Nissl stained sections of the SOC were provided. Furthermore, in a review article (Moore, 2000) it was again noted that there was no apparent MNTB in the human SOC. Here, it was unequivocally stated that the MNTB “cannot be identified in over 70 human brainstems” and that small clusters of NTB-like neurons were observed in the fetus. In a textbook chapter (Moore and Linthicum, 2004), the MNTB was noted to be vestigial in humans and to consist of only a few scattered oval cells. The MNTB was not labeled in their MAP2 stained section of the SOC.

Qualitative immunohistochemistry was used by Bazwinsky et al. (2003) to study the human SOC in three subjects (the number or spacing of sections was never stated; section thickness = 50 μm). These authors described only scattered CB-IR neurons in the region ventromedial to the MSO and claimed they could not further characterize the neurons in this region. They conclude: “In our material, we could not identify a coherent nuclear region that might correspond to the MNTB of other mammals. Neither Nissl stained nor CB immunolabeling of brainstem sections provided evidence of the existence of this nucleus.”

Hilbig et al. (2009) examined Nissl stained brainstem sections (thickness = 20 μm) from bonobo, chimpanzee, gorilla, orangutan, gibbon, macaca, and human (*n* = 12). They examined every 10th tissue section (e.g., only 10% of the SOC) and described only a few scattered neurons ventromedial to the MSO in the human. Only a single, small low-magnification view of the human SOC was provided. Based on this study, these authors also denied the existence of the MNTB in the human brain. The authors further noted a decrease in the size of the LSO from orangutans to humans. They provided an estimate of the number of neurons in the human MSO (3,891) and LSO (1,980) but indicated there were no MNTB neurons. Interestingly, they also provided photographic evidence of a single fusiform neuron in the area ventromedial to the MSO surrounded by a perineuronal net, although the methods use to label this neuron were not provided.

Reviewing all these studies of the human SOC, we found few figures illustrating the region ventral and medial to the MSO that had sufficient resolution to actually identify or negate the claims made about the existence of the MNTB. It was typical in these studies for only every 10th or 20th tissue section to have been examined without any sort of quantitative analysis. Thus, the conclusion that the MNTB does not exist in human was made from examination of less than 10% of the SOC. Regardless, it is apparent from these limited studies of the human SOC that a number of globular/round neurons are indeed present in region ventral and medial to the MSO cell column. We declare that these studies have not provided adequate evidence to support their conclusion.

There is no doubt that the MNTB is the major source of directly sound driven synaptic inhibition in the SOC affecting all major nuclei (LSO, MSO, SPN/SPON) and the LL (citations see above) which in turn send significant projections to next level within the ascending auditory pathway, the inferior colliculus in the auditory midbrain. The fact that early in development MNTB neurons release GABA (with some co-transmission of glutamate; Gillespie et al., 2005; Noh et al., 2010) but after hearing onset release almost exclusively glycine (Kotak et al., 1998), relates nicely to the function: this circuit requires speed and precision (Moore and Caspary, 1983; Park et al., 1996). The GBC pathway with its comparatively thick axons (Morest, 1968), axonal peculiarities (Ford et al., 2012) the calyx of Held synapse with its morphological and physiological synaptic specializations (von Gersdorff and Borst, 2002) and the tendency to directly target the post-synaptic soma rather than dendrites (Clark, 1969; Cant, 1984; Kapfer et al., 2002) provides temporally secure and very fast transmission from the CN to the MNTB allowing for a basically 1:1 input:ouput relation (Mc Laughlin et al., 2008; Englitz et al., 2009). This results in fast and precise glycinergic transmission to the target nuclei in SOC and LL. Moreover, the post-synaptic properties in particular of MSO and LSO with almost unmeasurably low input resistances (only a few MOhms) ensure unusually short temporal integration times on the post-synaptic side. The post-synaptic effects of the glycinergic inhibition of the MNTB depend on three parameters. (1) The post-synaptic properties, e.g., membrane time constant and temporal integration properties (Magnusson et al., 2005; Couchman et al., 2010; Mathews et al., 2010; Scott et al., 2010); (2) the interactions with other, excitatory inputs and (Pecka et al., 2008; Myoga et al., 2014); (3) the frequency domain [(a) within the phase-locking domain below or above 2–3 kHz and (b) the animals need and ability to process interaural time differences for localizing low-frequency sounds – these two aspects co-vary but are not identical; Grothe and Pecka, 2014].

The most prominent of the MNTB projections is directed toward the LSO. This projection was also the first to be described functionally and studied developmentally (Moore and Caspary, 1983; Kandler and Friauf, 1995). As far as we know today, this circuit applies to all non-aquatic mammals (there the anatomy and, even more, the functionality of the SOC is more or less unknown and therefore not discussed in this review) and its one and only purpose is sound localization. For higher sound frequencies, the MNTB provides the inhibitory side of one of the most simple, although highly affective, neuronal computations known: a simple subtraction of the inhibitor effect driven by the contralateral side from the ipsilaterally driven excitation (via spherical bushy cells in the cochlear nucleus) that results in the well-known sigmoid interaural level difference functions and it has been found in every single mammalian species studied (reviewed in Grothe et al., 2010). In order to allow the inhibition to affect the onset of the ipsilateral, excitatory drive and to enable the circuit to completely inhibit LSO output if the sound arises from the contralateral side, the inhibitory pathway via GBC and MNTB needs to compensate for the much longer axonal distance (the LSO is in close proximity to the ipsilateral cochlear nucleus) and for the additional synapse in the MNTB. It seems apparent that this most prominent and among mammals universal function of the MNTB directly relates to the peculiar morphological and physiological specializations observed in this nucleus as well as its universal appearance in all mammals including humans (although during evolution ITDs became more relevant for us, as a low-frequency hearing mammal we are actually quite sensitive to ILDs with a resolution of sometimes below 1 dB; Grothe and Pecka, 2014).

Besides the need for a fast transmission and conductance from the contralateral side, the LSO also provides evidence for the need of extraordinary accuracy. Even bats, that do not hear low frequencies and do not use interaural time differences, where it is comparably safe to say that the function of the LSO is ILD coding and nothing else, the LSO neurons function on a millisecond by millisecond basis (Moore and Caspary, 1983; Park et al., 1996). At first glance this may be surprising. However, consider multiple sound sources that partially overlap in spectrum and time [e.g., in a group of bats flying in a cave, but similarly relevant for us (for instance at a cocktail party)]. Such overlaps (whether direct sounds or echoes) result in wired ILDs that rapidly fluctuate (Meffin and Grothe, 2009). Hence, the LSO must be fast enough to provide reliable information about the sometimes very short periods of unambiguous ILDs and temporal summation of ILDs over time need therefore to be avoided. In any case, the fact that the LSO processes ILDs with sub-millisecond precision creates an epiphenomenon: a rough ITD sensitive to the onsets of the sound envelope. Therefore it is not surprising that the same excitation–inhibition interaction that creates ILD sensitivity also provides a basic mechanism for processing ITD (Moore and Caspary, 1983). As far as we know, the low-frequency limb of the LSO seems to exclusively process ITDs based on the interaction of the two known inputs (Moore and Caspary, 1983; Tollin and Yin, 2005) and is almost certainly the basis for the widely known trough-type ITD sensitive cells at higher levels of the ascending auditory pathway (Kuwada et al., 1997; Siveke et al., 2006).

If we take into account that the early mammals after inventing the middle ear lived in a high-frequency world (“ultra-sound” is a very anthropocentric term since most mammals can hear far beyond 40 kHz and, at the same time, many do not hear low frequencies at all), and therefore relied on ILD but not ITD processing (review: Grothe and Pecka, 2014), it does not come as a surprise, that the ITD processing structure, the MSO, receives the same inputs as LSO, only complemented by mirrored inputs from the contralateral CN (excitation) and the LNTB (glycinergic). The MSO is even faster, meaning shorter membrane times constants and shorter synaptic currents (Magnusson et al., 2005; Couchman et al., 2010; Mathews et al., 2010; Scott et al., 2010), and with an even clearer spatial limitation of the inhibitory (MNTB and LNTB) inputs to the soma only (Kapfer et al., 2002; Werthat et al., 2008). Therefore it seems unlikely that the temporal aspect of the MNTB input does not matter, even if the *in vivo* results (which to date are indirect if it comes to the cellular mechanisms underlying ITD processing) are controversial. On the other hand, there is new evidence for even more peculiar morphological adaptations in the low-frequency GBC inputs to the MNTB in the form of an unusual myelination pattern deviating from the general assumption of structural similarity (Ford et al., 2012).

Less controversial is the fundamental role of the MNTB mediated glycinergic inhibition in temporal processing. The earliest evidence came from mustached bat homolog of the MSO that shows highly precise phasic on and off responses to contralateral sounds (Covey and Casseday, 1991). Later it was shown that these responses result from an interaction of a tonic SBC excitation and glycinergic, MNTB driven inhibition. Depending on the relative timing (in some cells the excitation is faster, in some the inhibition – note again that the additional synapse in MNTB is compensated for) either an off or on response results and can be turned into a sustained response by blocking the inhibition (Grothe, 1994). Later, similar interactions of contralaterally driven excitation and inhibition were shown in the rabbit, rat and mouse SOC (Kulesza et al., 2007). It is particularly prominent in the SPN/MNTB (here the MNTB inhibition competes with a precise onset input derived from cochlear nucleus octopus cells) and is believed to function in gap detection (Kulesza et al., 2007; Kopp-Scheinpflug et al., 2011).

Much less is known about the VNLL where, again, octopus cells from the contralateral CN interact with MNTB inputs (Spangler et al., 1985; Sommer et al., 1993; Smith et al., 1998). One function seems to be the creation of an inhibitory onset delivered to the inferior colliculus that has an unusual intensity independent fixed delay (Covey and Casseday, 1999).

Besides MNTB derived glycinergic inhibition there is a very similar, so far largely under-appreciated glycinergic inhibition driven by the ipsilateral ear, which seems to by and large mirror/compliment the contralaterally driven MNTB inhibition. In fact, it shows some comparable structural adaptations (GBC-input; large end-bulb of Held synapses – large terminals or calyx-like; Spirou and Berrebi, 1997; Spirou et al., 1998) and has, at least in the MSO, *in vitro* very similar features as the MNTB inputs (Grothe and Sanes, 1994). Besides this there are intrinsic connections in SOC that are GABAergic, many of them, however, acting via GABA-B receptors and, hence, rather modulating responses than being a main component of the precise spatio-temporal computations (Magnusson et al., 2005; Stange et al., 2013).

Taken together, the MNTB is the main source of precise inhibitory inputs targeting all prominent SOC and LL nuclei of the ascending auditory pathway. This glycinergic input contributes an essential component to all spatio-temporal computations known in the SOC. From this point of view, the idea of a missing MNTB in humans is neither plausible nor explainable.

Although novel sources for inhibition are not unthinkable and there is circumstantial evidence that other inhibitory neurons may contact the usual MNTB targets in a transgenic mouse model missing MNTB (Jalabi et al., 2013; Altieri et al., 2014), the extraordinary anatomical and biophysical specializations characteristic of the MNTB make it highly unlikely that other sources of inhibition could fully compensate for MNTB function.

Therefore, we propose consideration of the principle of parsimony. This principle states that, in consideration of historical homology, the explanation that requires the fewest assumptions and phylogenic transformations is most likely to be correct. As the MNTB has been demonstrated (without question) to exist in other primates (rhesus, baboon, bonobo, chimpanzee, gorilla, orangutan, gibbon; Bazwinsky et al., 2005; Hilbig et al., 2009; Kim et al., 2014), and exhibits rather unique structural and biophysical adaptations to serve its function, it seems most likely that this nucleus would also be present in the human SOC. To suggest otherwise would require that humans have miraculously developed, *de novo*, new mechanisms for instance for sound localization.

### The Human MNTB: The Evidence for its Existence

Richter et al. (1983, in a paper entitled “Is there a MNTB in humans?”) studied three human brainstems using light microscopy (section thickness = 20 μm) and three additional brains using electron microscopy (ultrathin sections). They were able to identify the MNTB within the trapezoid body, ventro-medial to the MSO and lateral to axons of the abducens nerve. Their Figure 2 demonstrated a well-stained and clear view of the SOC with the MSO and “MNTB?” labeled. The authors noted that the neurons of the MNTB were fewer in number but similar in morphology to those in feline. Furthermore, they provided evidence for calyx-like endings in the MNTB territory in both protargol-stained sections and in ultrathin sections. Glendenning and Masterton (1998) examined auditory nuclei in 53 different mammalian species including humans. They confidently identified the MNTB in their human subjects. Notably, their results indicated that humans were below average in terms of MNTB size among the species examined (although well above average in MSO size) and further that there were species with an MNTB relatively smaller than that in human (Mountain Beaver, Great Glider, Little Rock Wallaby, and Eastern Gray Kangaroo). Finally, the author’s noted that in the 53 species examined the only SOC nucleus ever absent was the MSO (mice ^2^ and hedgehog).

Kulesza (2008) identified the MNTB as a loosely packed collection of neurons within the trapezoid body medial and anterior to the MSO in a series of 12 human brainstems (age range 55–94 years) using the Giemsa stain and Golgi impregnations. In Giemsa stained specimens, every second or fifth tissue section (40 μm in thickness) along the entire rostrocaudal axis of the SOC was examined. The MNTB was found to span a rostro-caudal distance of nearly 4 mm and was coextensive (through the rostral medulla and caudal pons) with the MSO and to contain ∼3,600 neurons. The vast majority (85–91%) of human MNTB neurons were round/oval principal neurons (Kulesza, 2008; Kulesza et al., 2011). In silver-impregnated material, MNTB neurons generally gave rise to restricted dendritic profiles, confined to the nucleus; dendrites rarely extended beyond the fibers of the trapezoid body. A number of photomicrographs of the human MNTB were included in this manuscript: a low magnification view of the entire SOC, a high magnification view of the MNTB (Figure 5) and a schematic series of the SOC along the entire rostro-caudal axis. It was later demonstrated that ∼25% of human MNTB neurons were associated with *wisteria floribunda*-positive and chondroitin sulfate proteoglycan-immunoreactive perineuronal nets and that the majority of these ensheathed MNTB neurons (59%) were principal (round/oval) neurons (Schmidt et al., 2010).

In a subsequent investigation of 11 human brainstems with immunohistochemistry for calcium binding proteins, it was found that the majority of human MNTB neurons were CB-IR and positive for the Kv3.1b potassium channel (Kulesza, 2014). Additionally, MNTB principal neurons were associated with large, CR-IR punctate profiles and numerous examples of CR-IR axons in the trapezoid body were identified giving rise to complex terminals associated with MNTB principal soma. Approximately 34% of human MNTB neurons were associated with such CR-IR calyx terminals (Kulesza, 2014). Further, numerous examples of VGLUT1-IR or Rab3a-IR punctate profiles were found associated with MNTB neuronal somata, supporting the presumption that such CR-IR puncta are axon terminals (**Figure 1**).

Previous studies of calyx endings in the rat MNTB reveal that the number of calyx-derived terminals decreases significantly with age (Casey and Feldman, 1985, 1988). We suggest this may also be the case in the human MNTB; our study of calyx terminals in the human MNTB utilized subjects that ranged from 59 to 96 years of age. However, in our estimates of neuronal number in the human MNTB (*n* = 26; 3–94 years of age) we have not found evidence for significant loss of neuronal number (Kulesza, 2008; Kulesza et al., 2011; unpublished observations).

Additionally, it should be noted that clear illustrations of the human SOC and MNTB were provided in plate 21 of the Olszewski and Baxter (2014) Atlas and in the Paxinos Atlas (obex +16 mm and obex +18 mm; Paxinos and Huang, 1995), although they were labeled as medial periolivary cells.

The GBC in the human ventral cochlear nucleus are large, round/oval cells with an eccentric nucleus and fine, diffuse Nissl granules. The GBC are most numerous at the root of the entering auditory nerve fibers. In human, the GBC appear less numerous than in cat (Moore and Osen, 1979; Moore, 1987). The majority of GBCs are CR-IR and give rise to calyceal axons that traverse the trapezoid body and terminate in the MNTB as the calyx of Held. The existence of a significant population of GBC in the human cochlear nucleus has been known for some time. However, we recently found that 84% of GBC in the human VCN were CR-IR (Kulesza, 2014). Additionally, we found that 80% of human GBC were associated with dense assemblies of CR-IR boutons, resembling modified endbulbs. Indeed, we also observed numerous CR-IR axonal profiles leaving the VCN and entering the trapezoid body. Finally, such CR-IR axons were observed to converge on the SOC and within the MNTB and to give rise to complex, calyx-type endings in relation to MNTB principal neurons. Intracellular injections of physiologically characterized cells in cats indicate that GBC axons project to the contralateral MNTB and periolivary cell groups, while the MSO and LSO receive weak, in any input (Smith et al., 1991). Based on these observations, it seems that the main function of GBCs is to provide fast, excitatory input to the contralateral MNTB (and ipsilateral LNTB) via large calyx-type endings. The identification of a prominent GBC cell population in the human VCN further supports the existence of a MNTB. Without an MNTB, GBCs would have had to develop a new function in the human auditory pathway (again, violating the principle of parsimony).

To summarize, we have identified the MNTB in each of 63 human brainstems with no known neurological diseases. The human MNTB was found consistently in the rostral medulla and caudal pons within the axons of the trapezoid body and ventromedial to the (easily identified) MSO. The vast majority of neurons in the human MNTB are round/oval as has been described for numerous other species. Furthermore, we have shown that many of the principal neurons in the human MNTB are associated with CR-IR axons and calyx-type terminals arising from GBC in the ventral cochlear nucleus. This finding corroborates the ultrastructural findings of Richter et al. (1983) and indicates that these neurons receive the same major synaptic input from GBC as do MNTB neurons in other mammalian species. Finally, human MNTB neurons are associated with a number of other “MNTB specific” markers. Specifically, we have shown that many human MNTB neurons are associated with perineuronal nets and that the majority of these neurons are CB-IR and express the Kv3.1b potassium channel.

Furthermore, we suggest that investigations of the primate auditory brainstem provide evidence that support the existence of the MNTB in the human brain. Specifically, the MNTB in rhesus and baboon is located amongst axons of the trapezoid body (Strominger, 1978) and these neurons are IR for parvalbumin and calbindin and are associated with large SYN+, CR or VGLUT1 positive calyx endings (Bazwinsky et al., 2005; Kim et al., 2014). Further, rhesus MNTB neurons are Kv3.1b positive and are associated with perineuronal nets (Hilbig et al., 2007). Indeed, we find that human MNTB neurons are similar in these regards.

Finally, it should be noted that the nuclei of the SOC are notorious for interspecies variation and their form depends on the auditory/frequency needs of the animal. The MSO is generally larger in species with excellent low-frequency hearing (Glendenning and Masterton, 1998). These authors further propose that the size of the LSO is directly related to the hearing range of the animal and that the LSO and MNTB co-vary in size. Thus, the size of the LSO and MNTB are correlated with the width of the audible range of the animal and it is suggested that hypertrophy of these nuclei are involved in broad band and/or high sound frequency reception (Moore and Moore, 1971; Heffner and Masterton, 1990). We have demonstrated that the human LSO contains 5,600 neurons while the human MNTB contains 3,600 (Kulesza, 2007, 2008). The numbers for the LSO are larger than counts provided by others (Hilbig et al., 2009; 1,980 LSO neurons; Moore and Moore, 1971; 2,500 – 4,000 LSO neurons). We attribute these differences in neuronal number to the number of tissue sections sampled and/or counting strategy. Regardless, we believe that our estimates of neuronal number in the MNTB (and LSO) reflect the reduced range of frequency sensitivity in human and the obvious relationship of the MNTB and LSO.

## Conclusion

Recent evidence clearly indicates the existence of a prominent MNTB in the human brainstem. The missing MNTB is a myth like that of the incisive bone before von Goethe (1830). Although for instance Vicq d’Azyr (1784) clearly showed its existence, the missing incisive bone was taken as the difference between the human anatomy and that of the animal kingdom. Similarly, calyces of Held as well as neurons at the right anatomical location for the MNTB had been described decades ago. Nevertheless, the absence of the MNTB in humans only seemed to have had, unconsciously, some sex appeal. Moreover, and conceptually more importantly, the role of glycinergeic inhibition in the SOC and MSO in particular was long ignored (note that the first evidences came from physiology and anatomy in the 60ies; Clark, 1969; Goldberg and Brown, 1969) but still leads to heated debates and some people acknowledge its there but “serves no function” (Portfors and von Gersdorff, 2013), although it would create clear problems concerning the phylogeny of the SOC with the MNTB as the major hub for inhibition involved in sound localization as well as temporal processing (Grothe, 2000; Grothe and Pecka, 2014). Regardless, the story of the human MNTB illustrates how difficult it is even for scientists to look with an unbiased eye.

## Conflict of Interest Statement

The authors declare that the research was conducted in the absence of any commercial or financial relationships that could be construed as a potential conflict of interest.

## Footnotes

1 Arguably, aquatic hearing may make certain structures obsolete because of the different physics of sound in water.

2 The reader should additionally refer to the description of the mouse MSO by Ollo and Schwartz (1979).

## Abbreviations

CB: calbindin
CR: calretinin
GBCs: globular bushy cells
ILDs: interaural level disparities
IR: immunoreactive
ITDs: interaural time disparities; LL, lateral lemniscus
LL: lateral lemniscus
LNTB: lateral nucleus of the trapezoid body
LSO: lateral superior olive
MNTB: medial nucleus of the trapezoid body
MSO: medial superior olive
SOC: superior olivary complex
SPN: superior paraolivary nucleus
VNLL: ventral nucleus of the lateral lemniscus
